# Abstract of the Proceedings of the Buffalo Medical Association

**Published:** 1863-02

**Authors:** J. F. Miner

**Affiliations:** Secretary


BUFFALO

Medical and Surgical Journal

VOL. II. FEBRUARY, 1863. NO. 7.

### ORIGINAL COMMUNICATIONS.

ART. I.—*Abstract of the Proceedings of the Buffalo Medical Association.*

TUESDAY EVENING, January 6th, 1863.

Prof. James P. White, President, in the Chair.

The minutes of the last meeting were read, eliciting a spirited discussion, Dr. White objecting to their adoption on the ground that he had been reported in full in a defense of his friend Dr. Sayer, and other physicians, while the attack—the cause of his defense—had been suppressed, thus making him appear in an unfair position.

*Voted*, that the minutes be corrected as suggested by Dr. White, and adopted.

"PREVAILING DISEASES."—*Dr. Rochester* reported diphtheria, measles and scarlet fever; scarlet fever and measles epidemically. Had no cases of these diseases, of particular interest to report at the present time.

*Dr. Wyckoff* reported scarlet fever as prevailing epidemically, and gave in brief, the circumstances of attack in several instances.

*Dr. Eastman* had seen scarlet fever, and erysipelas, accompanied by high inflammatory action; and also another disease, apt to prevail when erysipelas is prevalent, viz: puerperal fever. Had seen several cases, one of which proved fatal.

*Dr. White* would concur with the view that these diseases prevailed at the present time in the City epidemically; has also seen several cases of croup in connection with the diseases mentioned.

*Dr. Lockwood* had observed scarlet fever in remarkable differences as to severity of attack. A child on North Division street was taken with it in form. Nine days after a neighboring child, living next door, in very malignant type. A sister of this patient had slight attack, without any eruption; while a relative who was visiting them from Boston, had well marked diphtheria. First case, scarlet fever; second, scarlatina maligna; third, simple angina; fourth, diphtheria. Regarded this as a very interesting and instructive complication, showing the different varieties of the disease, and also its apparent relationship with diphtheria. The mild case of simple angina was followed by anasarca, one of the common sequelæ of scarlet fever.

*Dr. Rochester* reported a case of *masked* pericarditis. A vigorous young man, twenty-one years of age, was brought to the Hospital of the Sisters of Charity December 10th, laboring under acute mania. Nothing could be learned of his previous history, except that he had been ill for about a week; was delirious from the first, and had laterly become violent. Failing to discover cerebral disease, the intra-thoracic organs were examined. Marked and extended dullness was found in the precordial region The impulse of the heart could neither be seen or felt. The heart sounds were distant and muffled, and not distinctly separable. The pulse was slow, feeble and intermitting; the extremities were cold, and the prolabia livid. Physical exploration indicated an abscence of pleuritic effusion.—Two grains of opium were ordered to be given, and repeated every four hours, until the patient should become quiet; also iodide of potassium five grains, three times daily, and a blister 4 by 4 over the precordia.

December 11th.—The patient has taken, altogether, six grains of opium, has slept well, and is perfectly calm and rational. He ascribes his illness to a severe "strain in the breast from lifting a barrel of flour." The patient is now convalescent; the pericordial effusion is greatly diminished; the heart sounds are superficial and recognizable, while the pulse has increased in frequency and has entirely lost its intermitting quality. The patient was confined to his bed for twenty days; took two grains of opium every night for one week; had one blister over the precordia, and took iodide of potassium, as at first directed, during the whole period. He is now able to sit up during the greater part of the day, and is taking two grains of quinine every eight hours.

Dr. Rochester remarked that he reported the case as illustrating the great importance of examining the precordia in every case of recent insanity. without apparent cause. There was nothing to attract attention to this region, except the lividity of the nails and lips, and the unusual slowness and irregularity of the pulse—phenomena often ascribed to cerebral and hepatic disorders.

*Dr. Strong* remarked: "Mr. President, it has been intimated to me that a report of my doings and observations, while in the Army service, would be acceptable to the Association. With the best disposition to add to the interest of the meeting, I regret that I have kept no such diary or record as would be of great profit or interest to the Association. But, if acceptable, I will make a few extempore observations of what I saw, and heard, and did.

*First, as to the Locality.*—Frederick City, where most of my time was spent, is an old city of some ten or twelve thousand inhabitants. The most remarkable phenomenon which attracted my attention, after getting slightly acquainted in the city, was pertaining to the *personnel* of the profession there. It was the fact, that there are in that small population, two octogenarians, yet in active medical practice, neither of whom, whether regarded physically or mentally, would be taken to be more than sixty years old.

I had the honor, (and I regarded it as not a slight one,) to be called in consultation with one of these veteran practitioners in the case of a very prominent citizen, who was seized suddenly and very severely, with what was regarded by the family attendant (a practitioner of some ten years,) as congestive apoplexy. I regarded the attack as epileptiform in character. The notable feature of the consultation was, that while the family attendant was in favor of the most active depletory measures, venesection, &c., the octogenarian demurred, and advised diffusive stimuli, anodynes, &c. indicating full knowledge of, and accord to, the more recent views of the disease, and its requirements. The latter view prevailed in the consultation, and the resultant recovery justified its correctness.

The younger man had not outgrown the antiquated theory of congestion and what it demands, while the "*ancient*" had kept well abreast, with advancing and error-exploding medicine. It was a rare phenomenon.

In reference to military observations and experience, I suppose I have had about the usual admixture of impressions that were pleasant, and those that were otherwise; usual, I mean for those that have gone into the service, or gone to be observers, with their eyes open to see defects, when existing, and with an earnest desire and purpose to make or suggest improvements.

As regards the *personnel* of the Surgical Staff of the Army, with which I have come in contact, many of them were gentlemen of high professional character—ornaments to the profession and worthy of the noble service to which they are called. Some were men of mediocre capacity. And other some, were distinguished, I judge, by a somewhat violent eruption of gilt buttons and shoulder straps.

The hospitals of Frederick were occupied mainly by three classes of patients. First: Those that were sent from the army, ailing or sick. Second: The products of the battles of South Mountain and Antietam, who were not so severely wounded as to preclude their immediate removal to a point so distant as Frederick, and which constituted a class of primary cases. Third: A class of secondary cases, of a grade of severity generally to forbid immediate removal to Frederick, but not so severe when wounded in limb as to call for early amputation, or other operative means; cases in which the attempt was to be made to save the wounded member. Of these classes I will not remark at this time upon the first class, or those that were sick, farther than to indicate the diseases—which were for the most part diarrhœa, chronic or acute—camp fever, typhoid fever, camp rheumatisms and neuralgias—mostly, by far, the products of the ill-starred Peninsular campaign.

Neither will I remark upon the second class of cases, farther than to say they were generally minnie ball wounds, that for the most part required simple dressing, rest, time and patience, for recovery; cases in which the bone, if struck, was not so severely damaged as to involve the idea of removal or exsection.

Of the third class I would remark, that while I accord to most of the operations which I have witnessed, whether of amputatian or exsection, the praise, that they were skilfully, many times beautifully done, and while I accord to the good judgment generally evinced as to the length of time given to the attempt to save the limb and to avoid operative measures, I should not be true to my convictions, did I omit to say that there is far too generally in military surgery, a failure to appreciate the value of certain preliminary treatment in this class of cases.

Whether it arises from old views of the incompatibility of inflammation with tonics or stimulants, or from defective views of the powers and effects of quinine and opium, or from whatever source, in my judgment it is far too common that those articles, with at times alcoholic stimulants, are omitted during the stage of extensive suppuration, which attends this class of injuries.

Not that they are wholly ignored when the vital powers are palpably waning; nor that they are excluded in the immediate prospect of, or immediately subsequent to, operative measures. They are then frequently and assiduously resorted to; but if withheld till then, in these secondary cases, it proves generally wasted ammunition, the suppuration having been unrestrained in degree and unchanged in quality.

It can need no argument, methinks, to prove that an extensive suppuration from bullet wound, in whatever location, furnishes all the conditions which indicate the most potent supporting agencies known to our art. The drain upon the blood and the draft upon the nerve force is just such, it would seem, as to make an *early* and a *constant* administration of quinine and opium and whiskey, or its equivalent, with free alimentation, in any case in which there is *the least doubt of the unaided powers being equal to the shock*, an imperative necessity. It even seems a truism that should not require to be repeated or dwelt upon: and yet my observation, while in army service, impresses me that it is lamentably ignored in practice.

These views were acted upon in the hospital in which I had charge, and that with the happiest effect. Cases which in consultation had been consigned to the operating table have been saved from the necessity. Other cases which had been thought to be almost inevitably fatal, have resulted in recovery. It was quinine 2 grs., and opium 1 gr., every 4 hours, which did the work.

I should much deprecate being thought to arrogate any claim to originality, or to assume any claim to superior knowledge in presenting these views. Theoretically I believe they are almost universally accepted. Nothing but the conviction gained from considerable observation, that they are not sufficiently heeded in practice, could justify the free strictures here made As illustrative of the views here presented, I was informed of the record at one hospital, being twenty thigh amputations in secondary cases, and not one recovery.

I might take much time in reciting interesting cases of operations that occurred to me. I will now refer to but one. The wound was by ball, which struck the point of the right olicranon, and that of the internal condyle. The resulting disorganization in the course of four or five weeks, involved the whole humero ulnar articulation, denuding the whole articular surface but leaving unaffected the articulation of the radius with the external condyle, and with the ulna. Left to itself it became evident that soon the arm would have to be sacrificed to save life. The patient was kept steadily supported for weeks by quinine and opium, and then nearly the whole humero-ulnar articulation was sawed out, leaving intact the radial articulation. The result was a most agreeable one, leaving the forearm in a semi-flexed position, with good use of the fingers and good supination and pronation.

I may take occasion hereafter, with your leave, Mr. President, to report other cases that may prove of interest, but will occupy no more time at present.

*Prof. White* made remarks upon placenta prævia, alluding to the plan of artificial detachment of the placenta, suggested by Dr. Simpson, who inculcates the practice of separating the after-birth from its attachments in cases in which turning cannot be had recourse to. The oldest plan advocated by Dr. Radford, of Manchester, was to retain the fœtus and continue the gestation as long as possible; inclined to favor this plan of treatment, as much more safe both for the mother and child; objecting to the plan adopted by Dr. Simpson, that it predetermined the death of the child, while it did not improve the chances of the mother. Dr. W., related the particulars of two cases where a tampon controlled the hemorrhage until dilatibility of os-uteri allowed of turning and delivery with safety to mother and child. Described his manner of making and using a tampon which he insisted upon as important in the treatment of these cases, regarding it as the only means of controlling hemorrhage and thus saving the lives of both parent and offspring. Spoke of the objections which had been made to the use of the tampon and showed them as without good foundation.

The ground of Professor Simpson's plan or the main argument in its support seemed to be, that fewer cases of spontaneous detachment or expulsion of placenta previous to the birth of child, proved fatal to mother than after the operation of turning, and this had suggested the plan of making the separation of placenta a means of treatment. The statistics showing this include turning from all causes, and on this account are objectionable in determning this point. Spoke of the difference in the spontaneous separation of placenta by uterine contraction and consequent closure of vessels, as compared to artificial separation without contraction, and explained the risks of the latter and the comparative safety to the mother of the former.

Dr. White replied to inquiry by Dr. Wyckoff as to the proper time to retain the tampon, that it may be removed the next day if the hemorrhage had been arrested, and that for the time it will suffice, to be renewed when necessary.

*Dr. Wyckoff* spoke of the advantages of a T bandage in retaining the tampon, and the risk of leaving its support to the nurse; also his experience in the use of tampon and former method of introducing it. Regarded it as capable of arresting hemorrhage in almost all cases previous to delivery.

*Dr. Rochester* would enquire of the President his opinion of cotton wadding as material for tampon? Had formerly used the Kite-tail tampon, but has recently been using the wadding and likes it very well. Had treated two cases of utrine hemorrhage, using the cotton for a tampon, and was very much pleased with it, for that purpose.

*Dr. White* had used it and was very well pleased with its use: tares it in strips, tying them with tape to facilitate its removal.

*Dr. Wyckoff* announced the death of Dr. Alden S. Sprague, one of the ablest and most distinguished physicians of the city, during his period of active life. He gave a brief account of his life and of his last sickness and death; paying a fitting tribute to his memory.

Moved by Dr. Lockwood, and *voted*, to adjourn to meet with the Erie County Medical Society, to take appropriate action upon the occasion of the Death of Dr. Sprague.

J. F. MINER, Secretary.

